# A comprehensive overview of microbiome data in the light of machine learning applications: categorization, accessibility, and future directions

**DOI:** 10.3389/fmicb.2024.1343572

**Published:** 2024-02-13

**Authors:** Bablu Kumar, Erika Lorusso, Bruno Fosso, Graziano Pesole

**Affiliations:** ^1^Università degli Studi di Milano, Milan, Italy; ^2^Department of Biosciences, Biotechnology and Environment, University of Bari A. Moro, Bari, Italy; ^3^National Research Council, Institute of Biomembranes, Bioenergetics and Molecular Biotechnologies, Bari, Italy

**Keywords:** metagenome, shotgun sequencing, machine learning, metadata, disease prediction

## Abstract

Metagenomics, Metabolomics, and Metaproteomics have significantly advanced our knowledge of microbial communities by providing culture-independent insights into their composition and functional potential. However, a critical challenge in this field is the lack of standard and comprehensive metadata associated with raw data, hindering the ability to perform robust data stratifications and consider confounding factors. In this comprehensive review, we categorize publicly available microbiome data into five types: shotgun sequencing, amplicon sequencing, metatranscriptomic, metabolomic, and metaproteomic data. We explore the importance of metadata for data reuse and address the challenges in collecting standardized metadata. We also, assess the limitations in metadata collection of existing public repositories collecting metagenomic data. This review emphasizes the vital role of metadata in interpreting and comparing datasets and highlights the need for standardized metadata protocols to fully leverage metagenomic data's potential. Furthermore, we explore future directions of implementation of Machine Learning (ML) in metadata retrieval, offering promising avenues for a deeper understanding of microbial communities and their ecological roles. Leveraging these tools will enhance our insights into microbial functional capabilities and ecological dynamics in diverse ecosystems. Finally, we emphasize the crucial metadata role in ML models development.

## 1 Introduction

Human microbiome research has made significant progress in recent years, with a growing amount of metagenomic, metabolomic, and metaproteomic data that holds immense potential for hypothesis testing, meta-analyses, and disease diagnosis (Gilbert et al., 2018). However, several challenges hinder researchers from fully harnessing these resources, including the substantial time investments required, difficulties in accessing metadata, the demand for computational resources and bioinformatic expertise, and inconsistencies in annotation and formatting among individual studies (Pasolli et al., 2017).

Recently, several reviews and surveys have been published on the application of multi-omics approaches, particularly in the context of microbiome research. Marcos-Zambrano et al. (2021) focused on the application of machine learning (ML) techniques in human microbiome studies, covering topics such as features selection, biomarkers identification, disease prediction, and treatments. Hernández Medina et al. (2022) and Mathieu et al. (2022) overviewed how the latest microbiome studies harness the inductive prowess of ML and deep learning (DL) methods and considering how microbiome data peculiarities (i.e., compositionality, sparsity, and high-dimensionality)—necessitates adequate handling. Another noteworthy review article by Quince et al. (2017) emphasized best practices for shotgun metagenomic studies, discussed the identification and management of various technical limitations encountered during experimental approaches and provided an overview of implementing computational pipelines for shotgun data analysis. In a comprehensive discussion of experimental considerations for omics-based microbiome studies, Mallick et al. (2017) listed bioinformatics analysis tools tailored explicitly for metagenomics and metatranscriptomics and also touched upon the challenges associated with integrated multi-omic analyses. Nyholm et al. (2020) provided a perspective article that summarized the application of the holo-omics approach in biological research. They focused on holo-omics use cases in studies related to host-microbiota interactions, with an emphasis on exploring applications across various fields rather than engaging in a debate about available tools and methods. In a recent perspective Huttenhower et al. (2023) described how microbiome data sharing faces challenges due to its complexity and interdisciplinary nature. While best practices exist, they are not always widely adopted due to the effort involved. The need for microbiome-specific resources and recognition of data sharing efforts should be prioritized for progressing this field.

While these reviews and studies have significantly contributed to our understanding of microbiome research, there appears to be a noticeable gap in the public domain. Specifically, there seems to be a lack of comprehensive review articles that emphasize the critical importance of metadata in optimizing the implementation of ML and other advanced techniques within microbiome studies. Predictive models relying on artificial intelligence (AI) and ML tools have proven to be invaluable for gaining insights from the vast quantities of metagenomic data generated in laboratory. These tools also play a crucial role in unraveling the ecology and behavior of microbial taxa under study. AI and ML contribute to informed decision-making, effective management strategies, and conservation planning by providing a deeper understanding of microorganisms.

We aim to fill critical gaps in the existing microbiome research literature, with a specific focus on implementing machine learning (ML) techniques for microbiome classification while utilizing sample/raw metadata or disease metadata (pathological conditions) for each study and systematically reprocessing and reanalyzing the data. Unlike previous reviews, we highlight the importance of integrated metadata analysis, which involves discussing both experimental considerations (e.g., study design, sample collection, and sample processing steps) and bioinformatics considerations (e.g., managing diverse data types, assessing computational demands, selecting integration approaches, and analysis tools). We delve into the current landscape of metagenomic, metabolomic, and metaproteomic data analysis within microbial communities and concentrate on integrated metadata derived from metagenomic microbial community analyses. This review may be of interest to a broad range of researchers in the microbiome field, including those with expertise in ML, DL, and bioinformatics. We anticipate that our work will help to accelerate the development and implementation of advanced ML-based approaches for microbiome classification and disease diagnosis.

## 2 Exploring the diversity of microbiome data types and challenges in data analysis

### 2.1 “Omics” data types: understanding five distinct categories

Recent advances in next-generation sequencing (NGS) technology have enabled the generation of vast amounts of metagenomic data. Each of these data types provides unique insights into different aspects of the molecular world, and advances in high-throughput technologies and data science have made it increasingly possible to leverage all of these data types simultaneously (La Reau et al., 2023). Metagenomic sequences obtained with different sequencing strategies can be analyzed to answer a variety of questions: What is the relationship between the resolution of bacterial composition and the total number of obtained reads? To what extent do different sequencing methodologies selectively capture bacterial genera, resulting in exclusive identification by one strategy but not the other? To what degree do the sequencing approaches diverge in their capacity to explain relevant insights into specific experimental conditions? Moreover, other omics applications have been used to investigate the complexity in microbial communities, namely, metabolomics and metaproteomics. This wealth of data can be broadly categorized into five distinct types: shotgun sequencing, amplicon sequencing, metatranscriptomic data, metabolomic, and metaproteomic data.

#### 2.1.1 DNA-metabarcoding: profiling microbial communities

The most commonly used approach to analyze microbiota is DNA-metabarcoding (also known as amplicon-based metagenomics). In metabarcoding, samples are characterized using reads obtained through the selective amplification of marker genes, like the evolutionarily conserved 16S rRNA gene or the ITS region. 16S rRNA gene profiling allow us to characterize the taxonomic composition of prokaryotic communities while ITS (ITS1 or ITS2) has been suggested for fungi (Santamaria et al., 2012, 2018; Tangaro et al., 2021). Nonetheless, there are three main limitations in Amplicon Sequencing: (I) Taxonomic resolution and the ability to profile non-bacterial members of the community, such as Eukaryotes in the environment. The conservation of the 16S rRNA gene and the length of the amplicon product restrict the achievable taxonomic resolution. This means that certain closely related taxa may be difficult to differentiate based solely on the 16S rRNA gene sequence. Approaches based on the long reads sequencing (e.g. Oxford Nanopore and Pacific Biosciences), able to cover the whole 16S rRNA and ITS regions, are promising in reach species level taxonomic resolution (Johnson et al., 2019; Notario et al., 2023). (II) Inherent limitations in functional profiling: this approach attempts to estimate functional capacity using the 16S rRNA gene, it inherently lacks the ability to directly analyze the functional potential of microbes or microbial genes. Tools exist able to infer functional capabilities based on the taxonomic profiles such as Tax4Fun2 (Asshauer et al., 2015; Wemheuer et al., 2020) and the phylogenetic investigation of communities by reconstruction of unobserved states with PICRUSt (Langille et al., 2013) and PICRUSt2 (Douglas et al., 2020), but the accuracy and resolution of these predictions are limited. (III) PCR amplification and its effects: PCR-based marker gene surveys are vulnerable to a multitude of factors that can introduce errors and bias into microbiome studies. These factors, extensively documented in the literature (Nearing et al., 2021), encompass: undersampling, differential extraction contamination, storage bias, amplification parameters and quality of the starting template. Undersampling refers to the risk to obtain an incomplete representation of the microbial community due to limited sampling. Contamination from DNA introduced during laboratory experiment through reagents and equipment, known as contaminating DNA from reagents, is another concern. The sample storage conditions under which samples are kept can significantly impact the quality and quantity of DNA. The amplification parameters employed in PCR, including enzyme choice, annealing temperature, amplification time, ramp rates, and cycle number, can introduce variability and errors. Variations in the starting template concentration can also affect the outcomes of amplification. Furthermore, DNA properties such as GC content and secondary structure, known as template properties, can influence amplification efficiency. Errors may be introduced by primer mismatches or degeneracies, where primer sequences may not perfectly match target sequences. Polymerase errors during DNA polymerization in the PCR process contribute to the issue (Berden et al., 2022). Challenges also arise from chimeric reads, which are formed from hybrid sequences originating from different templates during amplification (Haas et al., 2011). Random errors, unpredictable in nature, can emerge during the PCR process, while systematic PCR errors may be associated with specific primer pairs or conditions. It's crucial to recognize that sequencing itself introduces errors, with Illumina sequencing posing particular challenges due to its imaging-based nature (Pienaar et al., 2006). These potential sources of error and bias has led to concerns about the accuracy, reproducibility, and potential contamination in microbiome studies (Gohl et al., 2016). Nonetheless, despite the need for PCR amplification, 16S rRNA gene profiling requires a relatively low number of sequenced reads per sample (~100,000) to maximize the identification of rare taxa. This makes it a cost-effective alternative compared to shotgun metagenomic sequencing (Peterson et al., 2021).

#### 2.1.2 Shotgun sequencing data: unveiling microbial abundance and functionality

Metagenomics experiments in the context of microbial communities employ a shotgun sequencing approach, which involves the isolation of DNA from the sample, its preparation for sequencing, and subsequent deep sequencing. Shotgun metagenomic (SM) data enable high resolution in estimation of taxon abundance from phylum (Sunagawa et al., 2013), to strain level (Scholz et al., 2016) within the original sample. In addition to taxonomic profiling, shotgun sequencing data is used for studying the functional potential of the human microbiome (Li et al., 2022). In the analysis of SM data, the sequencing depth serves as a crucial factor for understanding how it might affect the results. This impact is particularly evident when sequencing depth is insufficient, or the sample size is inadequate. A study by Li et al. (2022) reported that 15 million or higher depth as the optimal minimum sequencing to explore species level composition for metagenome-wide association studies (MWAS). The shotgun sequencing method has distinct advantages over targeted sequencing techniques, such as 16S rRNA gene sequencing. Shotgun sequencing is known for its relatively unbiased nature, making it a suitable choice for capturing the genomes of diverse species, regardless their phylogenetic origin (Lu et al., 2017). In addition, recent studies by La Reau et al. (2023) have revealed that shallow shotgun sequencing produced lower technical variation and higher taxonomic resolution than 16S rDNA sequencing at a much lower cost than deep SM sequencing.

There are several challenges and recommendations reported in SM sequencing: (I) Human DNA Contamination and Skewed Ratios: Challenges arise from shotgun sequencing approaches due to their propensity to generate reads in proportion to the relative concentrations of DNA within the sample. This often leads to an extremely skewed ratio of microbial to human DNA, resulting in human sequencing reads dominating within samples. For instance, stool samples typically consist of < 10% human DNA, whereas samples obtained from sources like saliva, throat, buccal mucosa, and vaginal swabs can contain more than 90% of reads aligned to the human genome (Lloyd-Price et al., 2017). (II) Removing host-derived DNA for accurate microbial analysis: Host-derived reads should be removed from the metagenomic data before downstream analysis by using available bioinformatic tools to avoid bias in microbial quantification (Pereira-Marques et al., 2019). (III) Distinguishing active from inactive microbial populations: A major limitation of SM is that this technique does not allow distinguishing between active (alive) and inactive (dead) microbial populations and whether the predicted genes are actually expressed and under what conditions.

However, some potential sources of bias are common to both SM and meta barcoding. For instance, DNA extraction methods can significantly impact the results. In addition, in the case of SM, it is crucial to consider the differences in sequencing total DNA through a PCR-free or PCR-enriched protocol. In this case, PCR bias is also common to both strategies. These biases can influence the resolution of bacterial composition, the selective capture of bacterial genera, and the capacity to elucidate insights into specific experimental conditions using different sequencing methodologies. Understanding and addressing these biases are crucial for accurate and reliable interpretation of metagenomic data (McLaren et al., 2019).

#### 2.1.3 Metatranscriptomic insights: revealing microbial activity

Metatranscriptomics is the study of the transcriptional activity of microbes and microbial populations, which is particularly useful for functionally investigate the gut microbiota. It is a powerful tool for understanding the active states of microbes, their genes, and the different expressed pathways, as well as for detecting and understanding the microbial role in pathological conditions. We can gain insights into the gene expression patterns of pathogenic microorganisms and their interactions with the host by examining the RNA transcripts present in a host microbiome. This information can aid in the early detection and diagnosis of infectious diseases, as well as in monitoring treatment efficacy and disease outcomes (Bashiardes et al., 2016).

However, there are some limitations to metatranscriptomic analysis in disease detection. First, the complexity of the microbial community and the varying abundance of different transcripts can make it challenging to assess their source from pathogenic or commensal microorganisms. Additionally, technical biases and limitations in sequencing technologies (i.e. reads length) may affect the sensitivity and accuracy of detecting low-abundance transcripts. Furthermore, the interpretation of metatranscriptomic data in the context of disease requires careful consideration of various factors such as the host immune status, sample collection techniques, and potential confounding factors. Standardized protocols for sample collection, RNA extraction, and data analysis are essential to ensure reproducibility and reliability of results.

Despite these challenges, metatranscriptomic analysis holds great promise for understanding host-microbe interactions in disease (Bashiardes et al., 2016), discover novel microbial interactions (Bikel et al., 2015), detect regulatory antisense RNA (Bao et al., 2015), and track expression of genes and determine the relationship between viruses and their host (Moniruzzaman et al., 2017). Advancements in sequencing technologies, bioinformatics tools, and data integration approaches will continue to enhance our ability to harness metatranscriptomics for accurate and informative disease diagnosis and monitoring (Shakya et al., 2019).

#### 2.1.4 Metabolomic signatures: unraveling interactions through metabolites

Metabolomics is an investigative approach focused on the analysis of small molecules (< 1.5 kDa), commonly known as metabolites, within various biological samples such as urine, serum, plasma, feces, and saliva. It is challenging to differentiate between features originating from microbes and those from the host or environment, so it is crucial to have clear links between these features and the corresponding microbial profiles from the specimen. These data become most valuable when closely connected to the corresponding microbial profiles from the source specimen. Also, this method aims to identify and characterize metabolites in these samples, thereby enabling the development of distinctive metabolic profiles for individuals or populations. These profiles are reflective of a complex interplay between genetic, environmental, and microbial factors.

Metabolomics encompasses two key approaches targeted and untargeted. Targeted metabolomics focuses on specific known metabolites, commonly used for validating biomarkers or studying the effects of interventions like drug treatments or dietary changes. It offers high sensitivity and precision but is confined to the predetermined metabolites on the target list. Untargeted metabolomics aims to identify and quantify all metabolites present in a sample, enabling the discovery of new metabolites, biomarkers, and pathways. While less precise than targeted metabolomics, this method provides a wider coverage of metabolites, shedding light on complex biological interactions involving genetic, environmental, and microbial factors. Distinguishing between features from microbes, the host, or the environment is challenging, requiring clear associations between these features and the respective microbial profiles from the specimen for accurate interpretation (Bingol, 2018; Yang et al., 2019).

A noteworthy illustration of this concept can be found in the examination of bioactive microbial metabolites, specifically short-chain fatty acids (SCFAs), which includes propionate, butyrate, and acetate. These SCFAs have been implicated in the development and progression of several diseases, including inflammatory bowel disease (IBD) and colorectal cancer (Storr et al., 2013). Additionally, there are other metabolites like bile acids, sphingolipids, and tryptophan derivatives, all of which exhibit evidence of microbial interactions and bioactivity within the gut environment (Mallick et al., 2019).

Recent studies by Muller et al. (2021) have demonstrated that it is possible to differentiate between individuals with IBD and those without, as well as distinguish between specific subtypes of IBD (ulcerative colitis and Crohn's disease) by employing ML pipeline and metabolic profiling techniques. This highlights the potential of metabolomics in contributing to our understanding of the underlying metabolic alterations associated with various diseases and conditions. Notably, these alterations include metabolites closely associated with critical microbial pathways like bile acid transformations and polyamines metabolism.

Noteworthy, obtaining, processing, and comparing microbiome-metabolome datasets from multiple studies is typically a cumbersome, extremely challenging, and time-consuming process. Initial challenges include downloading the data associated with each study, which are often missing or incomplete, and linking microbiome, metabolome, and metadata sample identifiers in each study. While sharing raw and/or processed metagenomics data is common and relatively standardized in terms of formats and online open-access repositories, metabolomics data is much less standardized and often not being shared in microbiome studies. Once all the raw data have been obtained, they need to be jointly re-processed, which often requires additional expertise or the use of a variety of bioinformatic methods. Making sure taxon and metabolite identifiers can be mapped and compared across datasets is another critical challenge and may require careful and tedious curation efforts. Schorn et al. have recently addressed some of these challenges by releasing a community resource for linking raw genomic/metagenomic data with metabolomic data (Schorn et al., 2021), yet, this resource requires proficiency in processing raw data sources and is targeted primarily at identifying and confirming novel links between biosynthetic gene clusters and metabolites (Muller et al., 2022). Regarding metabolomics raw data, the European repository MetaboloLights (Yurekten et al., 2023) currently contains 85 microbiome studies (out of 1,397, accessed 1/1/2024) and it is interesting to note how currently in the EMBL-EBI ENA (European Nucleotide Archive) repository are available 146,583 datasets, highlighting the limited amount of raw metabolomic data available (Yuan et al., 2023).

#### 2.1.5 Metaproteomics: revealing the proteome complexity

The gut microbiome, a highly intricate ecosystem comprising trillions of microorganisms, presents a challenge for conventional DNA-based approaches (Li L. et al., 2023). These methods often fall short in elucidating the functional aspects of the microbiome, unable to confirm whether predicted genes are actively expressed, under what conditions, or to what extent (Park and Graveley, 2007; Verberkmoes et al., 2009). Moreover, the viability and activity status of the microbial cells remain uncertain. Meta-transcriptomics (described above), although offering a solution by assessing RNA expression as an indicator of gene activity, encounters challenges related to the fate of expressed RNAs, ranging from protein production to degradation or epigenetic silencing (Holoch and Moazed, 2015; Yang et al., 2016). These limitations can be overcome by directly assessing proteins.

Addressing these limitations, metaproteomic emerges as a promising avenue, utilizing liquid chromatography–tandem mass spectrometry (LC-MS/MS) to delve into protein functions. Unlike DNA and RNA methods, metaproteomic directly assesses proteins, providing insights into microbial diversity and dynamic host-microbiota interactions in the human gastrointestinal tract. This technique aids in unraveling molecular mechanisms associated with both homeostasis and disease pathogenesis (Lee et al., 2017). In other words, metaproteomic is a large-scale characterization of the entire protein complement and was initially used to study the microbial function of environmental samples, like soil, activated sludge, and acid mine drainage (Long et al., 2020).

Despite its potential, metaproteomic faces challenges, notably in the depth of analysis due to the absence of a suitable database. Taxonomic diversity calculators, commonly used in gut microbiome studies, prove insufficient in assessing functional states. The need for a functional perspective becomes evident, as diversity alone does not necessarily correlate with the microbiome's functionality (Li L. et al., 2023).

Among metaproteomic studies, a mass spectrometry-based shotgun proteomics approach is employed. This technique involves the detection and identification of all proteins in a cell mixture without gel-based separation or de novo sequencing. Peptides resulting from enzymatic digestion of the proteome are separated by liquid chromatography and analyzed through tandem mass spectrometers. The resulting information is then compared against peptide databases derived from genome sequences. While shotgun metaproteomic has shown success in studies involving microbial communities with low diversity, adapting this approach to more complex environments, such as the human gut microbiome, remains technically challenging. This method has been demonstrated in few studies, including those focused on acid mine drainage systems, endosymbionts, and sewage sludge water. Indeed, in the ProteomeXchange (Vizcaíno et al., 2014; Deutsch et al., 2017, 2022) repository, 211 studies out of 31,443 (0.7%, data accessed on 1/1/2024) regards microbiome investigations. However, challenges persist, and further advancements are needed to overcome technical limitations in analyzing complex microbial communities (Verberkmoes et al., 2009). The pursuit of a comprehensive understanding of metaproteomics is strongly recommended, with a key reference available in Xiong et al. (2015). Erickson et al. (2012) described the simultaneous application of SM and metaproteomics to identify potential functional signatures in Crohn Disease (CD).

Table 1 summarizes the advantages, disadvantages, capabilities, and recommended use of metagenomic data types.

**Table 1:** Assessing metagenomic data types: advantages, disadvantages, capabilities, and recommended applications.

**Data type**	**Definition**	Capabilities^*^	**Advantages**	**Disadvantage**	**Recommended use**
Shotgun- metagenomics	Whole-genome sequencing of all genomes in a sample, including DNA from bacteria, fungi, viruses, and the host organism	• High resolution, • Moderate selectivity • High capacity	• Can identify all members of a microbial community, including novel and rare taxa. • Can be used to study gene expression and metabolic activity.	• Expensive, time-consuming, • May not be able to identify all bacterial genera at equal efficiency. • Difficult to assemble and analyze complex metagenomes. • May not be able to detect low-abundance taxa.	• Studying the diversity and composition of microbial communities, identifying new species and strains of microbes, • Investigating the functional potential of a microbial community
Amplicon- sequencing	Targeted sequencing of a specific gene or region of DNA from a sample	• Low resolution, • High selectivity • Medium capacity	• Can be used to target specific bacterial genera or genes. • Is relatively inexpensive and fast to generate	• Cannot identify all members of a microbial community • Biased toward certain bacterial genera	• Profiling the abundance of specific bacterial taxa in a community, Tracking changes in the microbial community over time, Identifying bacterial pathogens
Meta- transcriptomics	Whole-transcriptome sequencing of all RNA transcripts in a sample, including RNA from bacteria, fungi, viruses, and the host organism	• High resolution, • Moderate selectivity • High capacity	• Can be used to study gene expression and metabolic activity at a high resolution.	• Expensive, time-consuming, May not be feasible to identify all bacterial genera at equal efficiency. • Difficult to analyze, as it is not always clear which genes are being expressed by which bacteria	• Studying the functional potential of a microbial community, Identifying differentially expressed genes. • Investigating the response of a microbial community to environmental stimuli
Metabolomics	Identification and quantification of all metabolites in a sample	• Low resolution • Low selectivity • High capacity	• Can be used to study the metabolic activity of a microbial community • Can be used to identify novel metabolites.	• Cannot identify all members of a microbial community. • Biased toward certain metabolites. • Difficult to identify and quantify all of the metabolites present in a sample	• Studying the metabolic potential of a microbial community, Identifying biomarkers of disease • Analyze interaction between microbes and their environment
Metaproteomics	Study of the entire protein collection (proteome) of a microbial community	• Low resolution	• High-throughput, sensitive, quantitative	• Expensive, time-consuming, difficult to interpret results	• Study microbial communities, detect pathogens, and monitor environmental changes.

Capabilities^*^: - Resolution, The ability to distinguish between different microbes or genes; Selectivity, The ability to target specific microbes or genes for analysis; Capacity, The amount of data that can be generated and analyzed.

## 3 Machine learning for microbiome data analysis

In microbiome studies, there is a wide range of questions yet to be solved; these question follows how microbial communities and specific microbes within those community's cause, respond to, or contribute to disease. Do various diseases exhibit unique gut microbiome alterations? Are some conditions associated with pathogen intrusion, while others demonstrate a decline in beneficial bacterial populations? Can we pinpoint microbial biomarkers consistently enriched or diminished in a given disorder across diverse patient populations? Several recent studies have highlighted the advantages of implementing the ML pipeline on SM data to understand microbial taxa, identify signatures for disease identification and diagnose complex medical conditions, particularly for gut microbiome-related diseases. These studies demonstrate the following key benefits: (I) Improved Classification Accuracy to taxa associated with IBD: Mihajlović et al. (2021) employed a random forest (RF) model to classify Inflammatory Bowel Disease (IBD), achieving an average F1 score of 91%. This underscores the robust connection between IBD and the gut microbiome, showcasing how ML can enhance diagnostic accuracy in complex diseases. (II) Access the microbial taxa signature from SM data: Liñares-Blanco et al. (2022) generated a metagenomic signature using RF, effectively identifying IBD from fecal samples. The model achieved AUC scores of 0.74 and 0.76 for different IBD subtypes, Ulcerative Colitis (UC) and Crohn's Disease (CD), respectively, highlighting the utility of ML in subtype-specific diagnosis. Bakir-Gungor et al. (2021) utilized machine learning, specifically the RF method, to develop a classification model for Type 2 Diabetes (T2D) diagnosis and revealing that a subset of 15 commonly selected features had a significant impact on minimizing the microbiota required for T2D diagnosis, thereby reducing time and cost, showcasing the efficiency of ML in biomarker selection. (III) Biomarker discovery and patient subgrouping: Another study by Bakir-Gungor et al. (2022) aimed to identify biomarkers associated with human gut microbiota during IBD. Supervised and unsupervised ML models were employed to (i) aid IBD diagnosis, (ii) discover IBD-associated biomarkers, and (iii) Identify patient subgroups using clustering approaches. Random Forest outperformed other classifiers, shedding light on potential microbiome-mediated mechanisms of IBD and offering insights for microbiome-based diagnostics. Another study by Zeller et al. (2014) aimed to detect early-stage colorectal cancer (CRC) by employing metagenomic sequencing of fecal samples to identify distinctive taxonomic markers distinguishing CRC patients from those without tumors. CRC-associated changes in the fecal microbiome reflected, at least in part, the microbial community composition within tumors, indicating potential tumor-related host-microbe interactions. The analysis also revealed a metabolic shift from fiber degradation in controls to host carbohydrate and amino acid utilization in CRC patients, accompanied by increased lipopolysaccharide metabolism. IV) Geospatial Microbial Provenance: In a recent study Bhattacharya et al. (2022) implemented ML to enable geospatial microbial provenance. Researchers delved into the assessment of geographical specificity within environmental metagenomes. Primary objective was to discern unique microbial signatures that could be attributed to specific cities, relying on taxonomic classifications as the basis for differentiation. The outcomes of this comprehensive analysis unveiled a remarkable level of accuracy in pinpointing the origin of metagenomic data. The accuracy rates for classifying samples by city ranged impressively from 85 to 89%, while continental classification exhibited an even higher accuracy level, fluctuating between 90 and 94%. Leung et al. (2022) proposed an integration of metagenomics, metabolomics, and clinical data to classify enrolled participants based on their NAFLD (nonalcoholic liver disease) status and liver fat accumulation, and reaching an overall AUROC score of about 93%.

Also, ML offers a significant advantage over traditional statistics in the field of microbial ecology, where conventional statistical methods have been the norm for data summarization, hypothesis testing, and interpreting interactions within microbial datasets. The primary objective is to predict specific phenotypes, such as disease states or age, based on microbiome data. One fundamental distinction between statistical models and ML lies in their primary objectives: statistical models aim to describe and infer relationships between variables, whereas ML is tailored to optimize predictive accuracy on external datasets. To illustrate, supervised ML typically employs a learning step on a training dataset with labeled data patterns associated with specific outcomes, while a separate test dataset with unlabeled data is used to evaluate the model's performance. Finally, a validation dataset could be employed to further evaluate the obtained model, when unseen data (i.e. data not used neither for training nor for testing) are used. In contrast, statistical models primarily focus on understanding how values relate to outcomes, often without the need to partition the data for performance evaluation. ML possesses several advantages over classical statistics in microbial ecology research. It excels in detecting subtle variations in microbial community structure and can pinpoint particular bacterial taxa that play a pivotal role in predicting specific outcomes. Additionally, ML can model complex, non-linear combinations of bacterial count data and environmental parameters, which closely resemble real-world systems. This obviates the need for intricate data transformations or preprocessing, which can be challenging when dealing with molecular data.

Widening this aspect, ML approaches emerge as tool for multi-omics data integration. The aim of multi-omics (or integrative omics) approaches is to extract substantial evidence from large-scale data by identifying, classifying, and quantifying different biological molecules involved in complex structure, such tissues or microbial communities (Vailati-Riboni et al., 2017). An interesting application of multi-omics approaches was proposed by Monteleone et al. (2021) in which they linked microbiota composition and metabolites in Anorexia Nervosa (AN). This condition in characterized by weight loss/regain cycles. Authors characterize both the microbiota and the metabolome in the underweight and regain phases, identifying a perturbation in gut microbiota of AN female's patients compared to healthy ones, and an association to specific metabolites.

### 3.1 Utilization of publicly available microbiome data in research studies

The rapid advancement of NGS technology has led to an exponential growth in the volume of data housed within publicly accessible repositories like the GenBank by the National Center for Biotechnology Information (NCBI), the Metagenomic Rapid Annotations using Subsystems Technology (MG-RAST), the European Nucleotide Archive (ENA), and the DNA Data Bank of Japan (DDBJ), among others. These repositories are invaluable resources that store vast amounts of DNA sequences (Eckert et al., 2020). Utilizing these raw sequences, made available to the public, enables the application of cutting-edge ML and DL techniques for extensive data analysis. In this section, we aim to provide an insightful overview of the current trends in metadata analysis through the use of publicly accessible raw data and associated metadata.

Pasolli et al. (2016) conducted an extensive analysis of metagenomic data, involving 2,424 publicly available datasets. They introduced an ML-based framework for predicting microbiome-phenotype associations, focusing on species-level abundances and strain-specific markers. Cross-validation revealed strong disease prediction capabilities, especially when using strain-specific markers. Interestingly, including “control” samples from other studies in training sets improved predictions. *Streptococcus anginosus* was identified as a potential marker for general microbiome dysbiosis rather than specific diseases. This work advances our understanding of microbial dysbiosis and provides a publicly accessible software framework and data.

Duvallet et al. (2017) gathered data from 28 published case-control 16S rDNA amplicon sequencing gut microbiome datasets, encompassing 10 different disease states. Their objective was to explore whether consistent and disease-specific alterations in gut microbial communities could be identified across various studies of the same disease. Notably, some diseases, like colorectal cancer (CRC), exhibited an abundance of disease-associated bacteria, while others, such as IBD, were characterized by a depletion of beneficial bacteria. Specific conditions like diarrhea displayed substantial shifts in the overall microbial community, often involving numerous associated microbes, while most conditions showed only a few microbial associations this study identify unique patterns of dysbiosis shared across multiple disease states in the human gut microbiome, characterized by variations in the direction (i.e., the proportion of disease-enriched vs. disease-depleted genera) and the scope (i.e., the total number of genera showing differences between cases and controls) of disease-associated shifts. Pietrucci et al. (2022) investigated the possible association among gut-microbiome and Autism Spectrum Disorder (ASD) by using metabarcoding data from eight different project and 6 different geographical location. The applied several ML approaches and demonstrated their potential in overcoming limitation of classical statistical approaches and perform features selection in complex datasets.

Gupta et al. (2020) introduced the Gut Microbiome Health Index (GMHI), for assessing health status based on the species-level taxonomic profile of stool shotgun metagenome samples. GMHI evaluates the likelihood of disease presence, independently of clinical diagnosis, by comparing the relative abundances of microbial species associated with positive and negative health conditions. They implemented a mathematical index identified from a comprehensive dataset of 4,347 publicly available human stool metagenomes across various disease states. When they applied to large-scale dataset, GMHI effectively distinguishes between healthy and non-healthy groups, as compared to traditional ecological indices like Shannon diversity and richness. In Lam and Ye (2022) a network-based approach was implemented with aim to build a microbial association networks upon a subset of the Gupta et al. (2020) data. Additionally, they focused the more on analyzing diseases individually rather than a disease-agnostic approach, to better characterize microbial community traits in each disease. Lam and Ye (2022) by focusing on microbial community interactions in both healthy and diseased microbiomes, aimed at identifying factors for the stratification of disease states and the identification of potential microbial risk factors beyond individual species. Furthermore, to gain insights into community interactions across phenotypes, they also introduce a new metric called “module resilience” to study the retention of microbial community modules in microbial interaction networks.

Casimiro-Soriguer et al. (2022) performed a meta-analysis of 1,042 fecal metagenomic samples from seven publicly available studies. They applied ML pipeline based on functional profiles, instead of the conventional taxonomic profiles, to produce a highly accurate predictor of CRC with the aim to discriminate samples with adenoma, which makes this approach very promising for CRC prevention by detecting early stages in which intervention is easier and more effective. In addition, ML is used to extract features relevant to the classification, which reveals basic molecular mechanisms accounting for the changes undergone by the microbiome functional landscape in the transition from healthy gut to adenoma and CRC conditions.

Lugli et al. (2023) investigated the genetic diversity within bacterial taxa constituting the infant gut microbiome by utilizing the vast collection of publicly available shotgun metagenomic data and associated metadata from multiple global studies, encompassing infants from birth up to the age of 3 years. The extensive dataset, comprising 10,935 metagenomic profiles, enabled the identification of critical bacterial signatures within the infant microbiome, linked to distinct community-state types. Additionally, in the study metabolic reconstructions of these infant microbiomes shed light on the functional attributes of these predominant microorganisms during the early years of life, revealing potential correlations with health states from both metagenomic and metatranscriptomic perspectives.

Nelkner et al. (2023) conducted a meta-analysis using data from 16 primary studies, examining microbial communities in agricultural soils across Europe. They aimed to understand how European soil characteristics influence microbial community composition, particularly focusing on Thaumarchaeota members. Their analysis used publicly available metagenome sequencing data to assess microbial abundance at different taxonomic levels. This study highlights the significance of standardized metadata reporting and the benefits of open data sharing in the scientific community.

Key studies in microbiome research emphasize the significance of utilizing publicly available metagenomic data (Pasolli et al., 2016; Gupta et al., 2020; Lam and Ye, 2022; Lugli et al., 2023), which, when combined with metadata from different studies, facilitate the validation and confirmation of research findings. It also promotes data sharing, allowing scientists to build upon each other's work and develop comprehensive insights into complex phenomena.

### 3.2 Challenges to implementing machine learning

One key challenge is the interpretability of ML models, which often function as “black boxes” without clear mechanistic understanding. Interpretable ML approaches, such as deep forest algorithms and methods that incorporate prior knowledge like microbial interaction networks, are emerging to address this issue (Räz, 2024). The second barrier is the scarcity of large, high-quality, and correctly labeled microbiome datasets needed to train ML models effectively (Schloss, 2018). Additionally, ensuring data quality through techniques like deduplication, class balancing, outlier removal, and imputation is crucial. Lastly, selecting, evaluating, and tuning the right ML model for a specific task can be challenging, but a rich ecosystem of libraries and frameworks, as well as synthetic microbiome datasets, can aid in model development and benchmarking (Hernández Medina et al., 2022).

The challenges faced by ML in terms of metadata can be analogously compared to the complexities encountered in taxonomic annotation of bacteria, as discussed in the previous article by Mathieu et al. (2022). Definition and standardization of metadata: Over the past two decades, there has been a growing need for establishing not just standards for collecting and processing metagenomic data but also for developing well-defined methods for preparing metadata. This is essential to ensure the reusability of data and to train ML models for comprehensive and interdisciplinary microbiome analysis, as highlighted by Cernava et al. (2022). As bacterial species definitions are based on laboratory protocol and experiments, their relevant metadata including technical and analytical methods, must be well-defined and standardized in ML. The lack of clear metadata definitions can lead to difficulties in classifying bacterial species and organizing raw read data to perform effective statistical tasks. Data heterogeneity: Similar to the high DNA heterogeneity observed in bacterial species, metadata can vary greatly across different datasets and sources. This data heterogeneity poses challenges in integrating and comparing information when metadata standards are inconsistent. Moreover, considering we've only accessed a fraction of bacterial diversity on Earth, metadata used in ML may be incomplete and fail to capture the full spectrum of information needed for robust model training. Datasets may lack essential metadata attributes, making it challenging to build accurate models. Data representation: Just as metagenomic assembled genomes (MAGs) may not resemble complete genomes, metadata representation can be inconsistent or not following a standard format. This can make it difficult to interpret and utilize metadata for ML purposes. Taxonomy and classification: Similarly, integrating MAGs into metagenomic classifiers is complex due to their ambiguous taxonomy affiliations. In machine learning, associating metadata with specific categories or labels can be challenging when dealing with data that doesn't neatly fit into predefined classes. Integration with Models: Just as MAGs are not fully integrated into taxonomy, metadata may not always seamlessly integrate with ML models. It requires careful preprocessing and feature engineering to incorporate metadata effectively into the modeling process.

Yilmaz et al. (2011) introduced minimum information standard about metagenomic sequence (MIMS) and the minimum information about marker gene sequence (MIMARKS). Those are two widely used standards for reporting metagenomic and DNA metabarcoding data. These standards provide checklists of essential information for sharing data, such as the sample type, collection method, sequencing platform, and data processing steps.

In addition to MIMS and MIMARKS, there are a number of other standards that can be used to report specific types of metadata, such as the environmental package (E-Package): a standard for reporting environmental metadata associated with metagenomic samples (Logares et al., 2012) and the human microbiome project (HMP) data analysis pipeline: A standard for reporting metadata associated with human microbiome studies (Huttenhower et al., 2012) and microbiome quality assurance (MQA) a protocol for reporting quality control metrics for metagenomic and DNA metabarcoding data (Lassalle et al., 2018). The adoption of these standards makes microbiome data findable, accessible and, reusable for other researchers. This is essential for accelerating progress in metagenomics and DNA metabarcoding research (ten Hoopen et al., 2017).

The technologic advancements in instrumentation toward high-throughput and high-resolution methods in metabolomics, have supported the accumulation of big data across laboratories that needs a support regarding data and metadata deposition (Haug et al., 2017). The Metabolomic Standard Initiative (MSI) and COSMOS (COordination of Standards in MetabOlomicS) (Salek et al., 2015) are constantly supporting the definition of minimum standards in metabolomic data deposition by implementing the MSI Core Information for Metabolomics Reporting (CIMR) (Sumner et al., 2007). Moreover, COSMOS is actively engaging publishers to promote the requirements for authors to deposit metabolomics results, as is required for other “omics” disciplines (Salek et al., 2013). As an outcome of the COSMOS initiative, in 2014 the MetabolomeXchange database and repository was launched. It aggregates data from the major providers, namely MetaboLights (Yurekten et al., 2023), Metabolomics Workbench (Sud et al., 2016), and Metabolomic Repository Bordeaux, to facilitate the access and reusability of metabolomic datasets and associated metadata (Ferry-Dumazet et al., 2011).

Similarly to what happened for NGS data, proteomics and metaproteomics data release (both raw and processed) was initially driven by journals guidelines, and resulting in a lack of minimal associated metadata (e.g. experimental design, peptide identification and quantification, protein identifications and protein ratios) (Olsen and Mann, 2011). In this context, the ProteomeXchange (Vizcaíno et al., 2014; Deutsch et al., 2017, 2022) international consortium aims to overcome data and metadata deposition issues by exploiting the cooperation of primary [PRIDE (Perez-Riverol et al., 2021) and PASSEL (Farrah et al., 2012)] and secondary [PeptideAtlas (Desiere et al., 2006) and UniProt (The UniProt Consortium, 2023)] resources, bioinformaticians, researchers and also representatives from journals active in the field, and offering a framework for consistent and user-friendly data deposition.

### 3.3 Limitation of ML/AI application to microbiome data analysis

**Figure 1:**
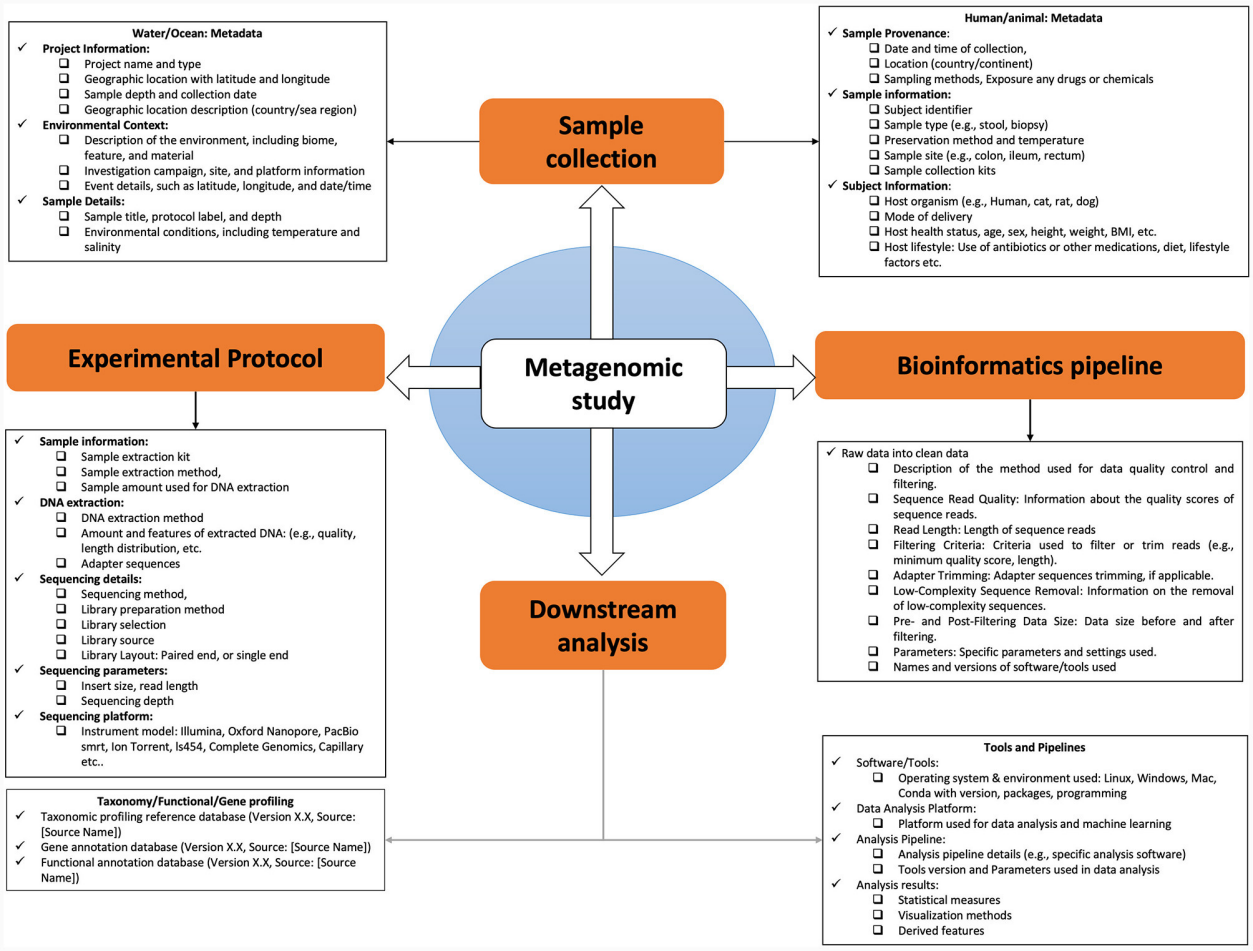
Comprehensive framework for metagenomic data documentation and metadata analysis.

Training by using a feature count table consisting of vectors composed of the relative representation of each taxon or MAGs in the sample is the most common approach to develop a predictive model (Figure 1), which is followed by normalizing the raw counts using an appropriate approach accounting for sparsity and data compositionality (Gloor et al., 2017; Casimiro-Soriguer et al., 2022). However, the implementation of ML comes with its own set of limitations, potential errors and common challenges associated with applying ML to this input data:

#### 3.3.1 Data quality and pre-processing

Due to the high dimensionality, sparsity, and noise of metagenomic data, a significant challenge arises during the normalization process to feed into the ML model. Non-biological zeros are a prevalent phenomenon observed in both 16S rRNA and SM datasets (Jiang et al., 2021). The abundance distributions of taxa are distorted by these zeros, which can be attributed to three distinct categories: biological, technical, and sampling zeros (Brill et al., 2022). Biological zeros correspond to actual zero abundances of taxa that do not exist in the microbiome samples. In contrast, technical zeros and sampling zeros are non-biological zeros with distinct origins. Technical zeros result from pre-sequencing experimental artifacts, such as DNA degradation during library preparation and inefficient sequence amplification driven by factors like GC content bias (Silverman et al., 2020). On the other hand, sampling zeros stems from limitations in sequencing depths. Addressing the intricacies associated with these zero categories is imperative for robust ML model construction. In addition, a typical dataset may contain a few hundred training instances but thousands of OTUs/ASVs (i.e., features); this large number of features can greatly challenge the classification accuracy of any method and compound the problem of choosing the important features to focus on.

#### 3.3.2 Biological complexity

The microbiome is variable between individuals and time. This biological variability can make it challenging to identify universal patterns or develop generalizable models (Kodikara et al., 2022; Vinciotti et al., 2023). Also, the taxonomic and functional variability of microbial communities can exhibit significant differences across different environments, making it difficult to establish consistent associations.

#### 3.3.3 Interpretability

Complex machine learning models, such as deep neural networks, might lack interpretability, making it challenging to understand the biological significance of the learned patterns as these models may not be able to generalize to new, unseen data (Linardatos et al., 2021). Interpretable models are often preferred in microbiome research to gain insights into the relationships between microbial features and expected outcomes (Bengtsson-Palme, 2020).

#### 3.3.4 Overfitting and generalization

Due to the high dimensionality of microbiome data, models may be overfitting to noise and contain many spurious correlations in the training data (Walsh et al., 2023). To prevent overfitting, we can use several techniques, such as early stopping, regularization, and data augmentation (Balestriero et al., 2022). Early stopping involves stopping the training process before the model has fully converged, while regularization involves adding a penalty term to the loss function that discourages the model from overfitting (Schmidt, 2023).

Imbalance dataset and cross-validation issues may lead to optimistic estimates of model performance. In this case recommended to use methods like stratified cross-validation techniques to account for class imbalances in microbiome datasets (Gou et al., 2020; Casimiro-Soriguer et al., 2022; Watson, 2022).

#### 3.3.5 Batch effects and confounding variables

Batch Effects are very common, and this often introduces systematic differences between the measurements of different batches of experimental such as sites/between laboratories, sample preservation protocols, storage conditions, DNA/RNA isolation methods and kits (Ling et al., 2022; Li Y. et al., 2023), sequencing methods can introduce batch effects, which may confound the true biological signals. Combining data from different batches without carefully removing batch effects can give rise to misleading interpretations of taxonomical classificational and ML model interpretations. Therefore, it is necessary to identify and remove the batch effects before proceeding to the downstream analysis and proper normalization and batch correction techniques are essential (Luo et al., 2010) and multiple approaches for batch effect removal have been reported (Alter et al., 2000; Benito et al., 2004; Ling et al., 2022).

Confounding Variables such as diet, medication, and lifestyle can influence the microbiome composition (Li Y. et al., 2023). Failure to account for confounding variables may lead to spurious associations (Al Bander et al., 2020).

Feature Selection and Dimensionality Reduction are used to face the sparsity of microbiome data issue, which makes it challenging to identify important features and patterns through the input data (Lee et al., 2023). Feature selection or dimensionality reduction techniques must be applied during model training.

#### 3.3.6 Model validation and reproducibility

Lack of independent datasets for validation, testing, or failure to reproduce results can undermine the reliability of ML in microbiome analysis (Rojas-Velazquez et al., 2024).

Pammi et al. (2023) reviewed the use of artificial intelligence in integrating “multi-omic” and compared metagenomics analysis approaches, highlighting the effectiveness of statistically equivalent signatures for feature selection and random forest modeling in achieving accurate disease diagnosis and biomarker discovery in colorectal cancer patients.

## 4 Understanding metadata: data about data

Metadata is “data about data” (Cernava et al., 2022) refers to contextual information associated with metagenomic experimental data offering a comprehensive understanding of the sample's background. In microbiome research, metadata's definition varies based on the type of metagenomic sample under analysis. For instance, metadata for a human gut sample will differ from that of an ocean sample, yet both serve to contextualize the data. Metadata plays a pivotal role in providing context by describing various aspects of the sample, including collection time points, geographical location, biome type, environmental or experimental conditions, and sample pre-processing steps (Leipzig et al., 2021). The structure of metadata can vary by study, but it typically includes features such as chemical data (e.g., pH, salinity), physical data (e.g., temperature, incident light), sample collection time points, host condition (disease/healthy), diet variations, antibiotic exposure, and geographical location (Nassar et al., 2022). Moreover, metadata should encompass information on sampling methods, sample size, and sample preparation techniques. Precise metadata annotation is crucial for detailing the sample source, tissue collection methods, environmental characteristics, and additional specifics like DNA extraction protocols, sequencing library preparation methods, and sequencing depth. In essence, metadata enriches metagenomic data by providing the critical context needed for analysis and interpretation in microbiome result (Nassar et al., 2022).

### 4.1 The significance of comprehensive metadata in microbiome research

**Figure 2:**
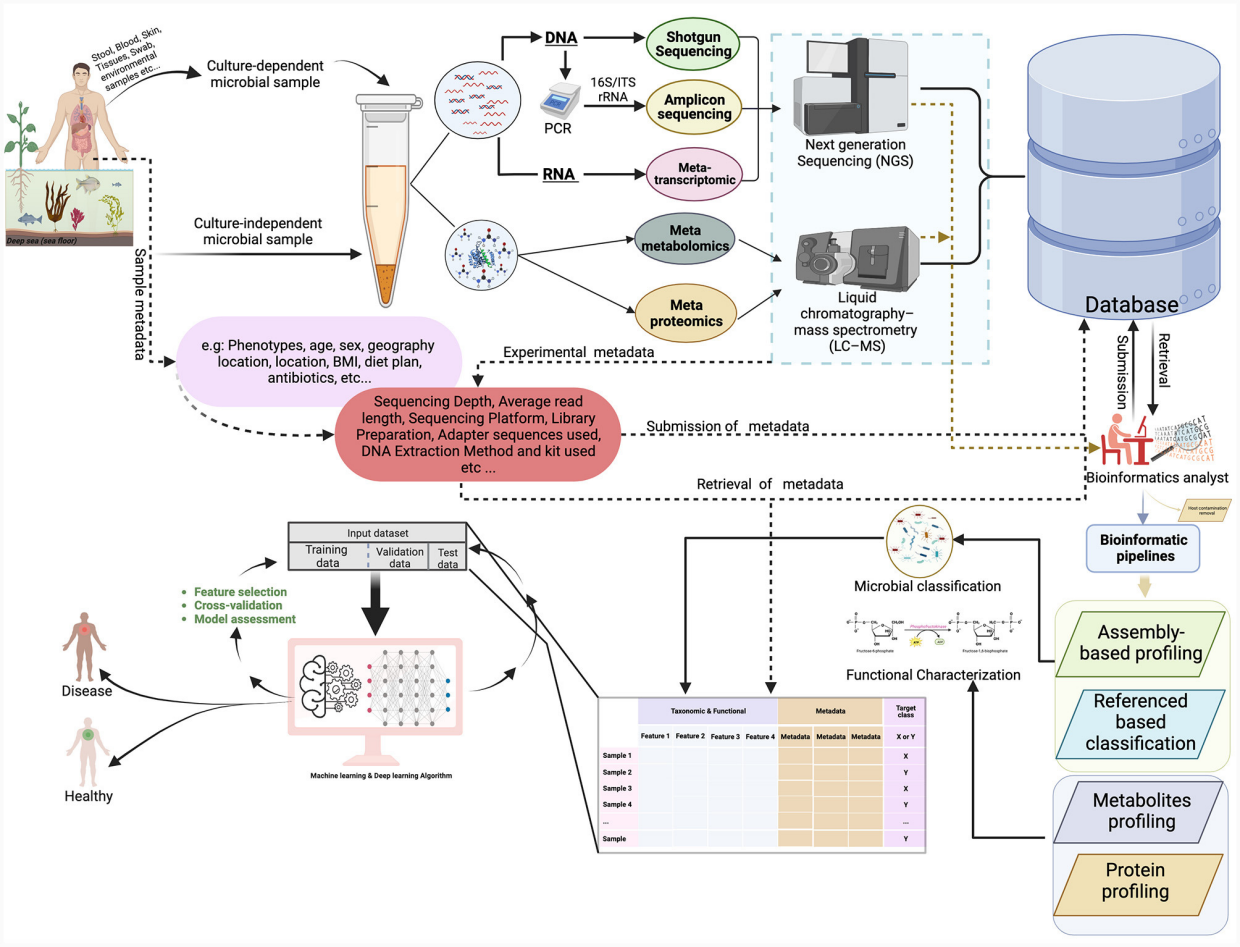
This figure provides an overview of the microbiome workflow for studying microbial communities using shotgun sequencing, 16S rRNA gene sequencing, metatranscriptomics, metaproteomics, and metabolomics. The figure illustrates the process of sample collection from various sites and then proceeds through different experimental procedures, bioinformatics pipelines, and ML analyses. The figure was created with https://www.biorender.com/.

The collection and utilization of various metadata elements in microbiome research are of paramount importance. These elements encompass a wide array of information, from the characterization of the microbiome's natural environment (ecoregion) to the specific host organism (host microbiome) and even human-made environments (engineered microbiome). For a microbiome study, metadata exists at multiple stages along the path from sampling to analysis of omics data as shown (Figure 2). This metadata falls into two main categories: assay metadata, which encompass technical details like machine type, assay date, and reagent kits, and biological metadata, which describe experimental aspects like sample conditions, exposure to drugs, animal housing conditions, or host genetic information. The absence of such information may affect downstream statistical analysis and even qualitative interpretation challenging or impossible.

#### 4.1.1 Sample metadata

Information about provenance and characteristics of the samples: when it was collected (e.g., date and time), where it was collected from (e.g., latitude, longitude, elevation/depth, site name, country, etc.), what kind of sample it was (e.g., soil, seawater, feces/stool), and the properties of the environment during collection (e.g., temperature, salinity, pH) or if sample is clinical then phenotypic condition (e.g., age, sex, disease state/normal) from which the sample was taken and the nature of the sample material itself all contribute valuable context to microbial studies (Wood-Charlson et al., 2020; Vangay et al., 2021).

#### 4.1.2 Experimental metadata

It is subjected to preparation steps for nucleotide sequence analysis or metabolome/metaproteome. Information about experimental preparation of the original sample (Gohl et al., 2016; Vangay et al., 2021). A sample could be split (aliquoted) and processed through multiple preparation methods; therefore, there could be multiple sets of preparation metadata for a single set of samples such as controlled or treated. For DNA sequencing preparation metadata include the type of DNA, extraction protocol, conditions used for sequencing (e.g., primers, library kits, sequencing instrumentation, and parameters), and where the raw sequence data sets are accessible. The information required to properly describe metabolome and metaproteome data are even more complex and workflows profoundly change according to the used platforms and technologies (Rechenberger et al., 2019).

#### 4.1.3 Data pre-processing metadata

Data about the properties and downstream processing of the raw reads data, including software/tools parameters and version. For example, if DNA sequences were generated, this could include the sequence properties (e.g., sequence lengths, sequences per sample, and total base pairs, total percentage of GC content, percentage of sequence duplication), quality control and filtering (e.g., sequencing depth, adapter trimming, quality trimming and filtering, dereplicating, and chimera sequence removal), assembly parameters (e.g., assembly tool, binning tool, and finishing strategy), reference genome used (version and source), gene annotation (e.g., gene calling tool and annotation database), and other processing parameters (Roy et al., 2018).

#### 4.1.4 Feature metadata

Data about features detected in the raw data, rather than about the samples themselves. For example, if amplicon sequencing was performed, feature metadata might include information (e.g., taxonomy, reference genome sequences with version information and source, and sequence identifiers) about the OTUs or ASVs generated in the OTU-picking or denoising algorithm, respectively. If metabolomics analysis was done, feature metadata might include information (e.g., mass spectrometry (MS2) fragments produced or candidates for identification) about the metabolites detected. Obtaining key metadata from sample collection to data analysis would greatly improve reproducibility. For metaproteomics, it might include identified proteins and related pathways.

### 4.2 FAIR data principles in metagenomics and machine learning

The FAIR Data Principles are a set of guidelines for making data more findable (F), accessible (A), interoperable (I), and reusable (R). These principles are important for both data sharing and machine learning, as they help to ensure that data is discoverable, accessible, and compatible with different machine learning algorithms and tools (Wilkinson et al., 2016). In the context of metagenomics and machine learning, the FAIR Data Principles can be applied to the following: Findability: Metagenomic data should be deposited in public databases, such as the NCBI Sequence Read Archive (SRA) or the European Nucleotide Archive (ENA). These databases provide unique identifiers and searchable metadata for each dataset, making it access the data they need. Accessibility: Metagenomic data should be accessible to researchers using standardized protocols, such as hypertext transfer protocol (HTTP) or file transfer protocol (FTP). This ensures that researchers can access the data regardless of their computing environment. Interoperability: Metagenomic data should be stored in a format that is compatible with different machine learning algorithms and tools. This allows researchers to easily use the data to train and evaluate machine learning models. Reusability: Metagenomic data should be released with clear and accessible data usage licenses. This consents researchers to reuse the data for their own research without having to concern about copyright or other restrictions (ten Hoopen et al., 2017; Vesteghem et al., 2020).

### 4.3 Metadata standardization: ensuring data accuracy

Despite the critical nature of metadata, metadata collection is often poorly standardized and error prone. Tabular formats (such as Microsoft Excel) continue to be popular options for metadata collection and record-keeping, yet freeform text entry without validation is prone to errors (e.g., misspellings, incorrect data, missing data, and inconsistent values) (Schloss, 2018). These issues can emerge within a single study and are even more likely across multiple studies. For example, with standardized metadata, experimental results from different labs can be grouped together for combined studies with a scope that can extend beyond what can be done from a single lab (Thompson et al., 2020). It also lays the foundation for researchers to quickly find previous experiments of interest to them. Situations may arise where obtaining precise coordinates for certain locations becomes a complex endeavor. These challenges can stem from various factors, including governmental restrictions imposed in specific countries or regions, intellectual property protection, or concerns related to data privacy and property rights. These issues are particularly prominent in datasets associated with potentially sensitive subjects, such as high levels of pathogens or antibiotic resistance genes (Serwecińska, 2020). In some cases, private landowners may be unwilling to disclose the exact locations of their facilities. They might wish to avoid negative associations with their business operations, especially in situations where their facilities are associated with research findings concerning pathogens or antibiotic resistance genes. Moreover, researchers in the industrial sector may be hesitant to make data on specific field sites publicly available. This averseness may be motivated by the fact that these sites are involved in testing new plant cultivars and breeding efforts. The proprietary nature of their work and the competitive landscape could drive this concern. In the realm of biological data and microbiome research, there is a growing awareness of the need to protect the collection coordinates of endangered species, including those listed on conservation red lists (Zhu et al., 2021). This keen concern is rooted in efforts to combat poaching and illegal collection of these species. As a result, there is an ongoing debate regarding how to balance the imperative of protecting these species with the need for scientific data sharing (Levesque, 2017). Lastly, governmental organizations may also have reservations about disclosing precise locations of sites deemed geopolitically important or contaminated. Such disclosures could have difficulties for national security, public safety, or environmental concerns.

### 4.4 Navigating metadata challenges in metagenome databases

#### 4.4.1 Lack of Metadata

One major limitation of existing public repositories and specialized metagenomic databases (e.g., NCBI, ENA, SRA, MGnify, MG-RAST, NMDC, QIITA) is the often incomplete and inconsistent metadata associated with metagenomic samples. Metadata it is frequently missing or inadequately annotated, making it challenging to perform cross-study comparisons effectively. Lack of Standardization: Metagenome databases suffer from a lack of standardized metadata. Metadata across different studies and databases may use varying terminologies, formats, and ontologies, leading to difficulties in harmonizing and integrating data for meaningful analysis. Difficulty of Metadata Annotation: Manually annotating metadata for metagenomic samples is a labor-intensive and time-consuming process (Kasmanas et al., 2020). While some efforts have been made to standardize metadata using controlled vocabularies and ontologies, these approaches are not always comprehensive or flexible enough to capture the diversity of sample origins, particularly in engineered environments (Cernava et al., 2022). Inefficient Sample Retrieval: Retrieving samples of interest from existing metagenome databases can be incompetent and challenging. The lack of standardized metadata and user-friendly search interfaces makes it difficult for researchers to select relevant samples based on specific criteria, such as host characteristics or environmental factors (Clark et al., 2022). Limited Cross-Study Comparisons: The inconsistent and incomplete metadata in metagenome databases hinder the ability to perform meaningful cross-study comparisons (Nassar et al., 2022). This limitation restricts the potential for meta-analyses and the discovery of patterns or associations that may not be evident in individual studies. Dependence on Manual Annotation: Many existing efforts to improve metadata quality rely heavily on manual annotation, which is not scalable to handle the exponentially increasing volume of metagenomic data. This limitation can lead to delays in data availability and the inability to keep up with the pace of data generation (Kasmanas et al., 2020). Complexity for Non-Bioinformaticians: Some databases that offer comprehensive metadata are not easily accessible to non-bioinformaticians. For example, metadata stored as *ExpressionSet* objects in R environments can create complexity for researchers who are not proficient in bioinformatics. Limited Support for Specific Environments: Hierarchical ontology relationships may not adequately describe diverse and specific environments, such as engineered environments. Existing controlled vocabularies and ontologies may lack the necessary granularity to capture the full range of sample origins. Inflexible Ontology Relationships: Some databases rely on hierarchical ontology relationships, which can be inflexible and may not accommodate the complexity and diversity of environmental descriptions adequately (Romano et al., 2011). The limitations of existing metagenome databases primarily revolve around the challenges related to metadata quality, standardization, and accessibility. These limitations hinder the full potential of metagenomic data analysis and the ability to perform comprehensive cross-study comparisons and meta-analyses. The development of automated methods for metadata extraction and more user-friendly interfaces is essential to address these limitations and unlock the full value of metagenomic datasets.

### 4.5 Root causes of annotation errors in public databases

Despite some notable progress in data-sharing policies and practices, accurate and reliable annotation of metagenomic data in public repositories is crucial for dry laboratory researchers and their subsequent applications. In public databases such as NCBI, European Nucleotide Archive (ENA) (Yuan et al., 2023), Sequence Read Archive (SRA) (Katz et al., 2022), MGnify (Richardson et al., 2023), MG-RAST (Meyer et al., 2008), and National Microbiome Data Collaborative (NMDC) (Wood-Charlson et al., 2020), the reliability of annotations heavily relies on the metadata provided by researchers during the submission of sequencing data. However, following are listed several root causes that have been identified that contribute to annotation errors within these databases. (i) User metadata submission errors: Researchers are responsible for submitting metadata that describes the characteristics of their raw/processed sequence, including the name of the model or host organism, pathological conditions (diseased/healthy), biomaterial provider, collection date and time, tissue or samples, developmental stage, and geographical location. However, if researchers make errors or inaccurately assign metadata, it can lead to miss-annotation of sequences and associated data. For example, if a researcher studying soybeans from soybean roots mistakenly assigns the organism's name as *Glycine max* instead of *Glycine soja*, all sequences tied to that metadata will be incorrectly labeled as *Glycine max*, leading to potential misinterpretation and inaccurate analyses (Nassar et al., 2022). (ii) Contamination errors in biological samples: During sample collection and processing, contamination from unintended sources can occur, resulting in the misidentification of organisms or genetic material. If such contamination goes unnoticed or unaddressed, it can lead to incorrect annotations in the public databases. For instance, if a sample intended for sequencing a specific organism becomes contaminated with genetic material from different organisms (usually microbials), the resulting sequences may be incorrectly labeled and associated with the wrong organism in the database (Schnoes et al., 2009). (iii) Bioinformatic tools inaccuracies can lead to erroneous annotations. Different bioinformatics tools and algorithms are utilized to process and annotate sequencing raw data. However, these methods can introduce errors or biases that propagate throughout the database. Imprecise algorithms or incomplete reference databases and versions can result in miss-annotations or missing annotations for specific sequences, further compromising the reliability of the database (Schnoes et al., 2009).

### 4.6 Challenges and debates in data release protocols: balancing recognition and access

Despite developments in data-sharing policies and practices, many genomic datasets remain restricted even after approval for public release. This conflicts with the terms of funding agencies, which support data dissemination for science and society progress. The lack of clear and comprehensive guidelines for data usage compounds the issue (Schnoes et al., 2009). Public domain data release protocols acknowledge the tension between unrestricted access and data producers desire for recognition through first publication rights. This conflict has led to multiple interpretations, fuelling an ongoing debate about how publicly available data should be used. The pressure to be the first to uncover significant discoveries can lead to data withholding until after publication, hindering broader dissemination (Tenopir et al., 2020). Even after publication, challenges persist, including time constraints in preparing data for sharing, legal and privacy considerations, and concerns about misinterpretation or misuse. Researchers often face difficulty locating the data they need, devoting up to 50-80% of their time to these obstacles (Eckert et al., 2020). Vangay et al. (2021) sustained identifying and addressing the root causes of annotation errors in public databases is essential for maintaining data integrity and ensuring the accuracy of downstream analyses and research applications. By taking into consideration of the factors that contribute to miss-annotations, efforts can be directed toward implementing quality control measures, improving metadata validation processes, enhancing contamination detection methods, and refining computational tools to minimize errors and improve the reliability of public databases.

### 4.7 Privacy concerns in metagenomics: uncovering personal information

The availability of open-access metagenomic datasets provides a valuable resource for studying health- and disease-associated signatures of microbial communities. However, an ongoing debate within microbiome research revolves around addressing privacy concerns to protection of personal information (Guccione et al., 2023). Franzosa et al. (2015) investigated the human microbiome by utilizing metagenomic codes. These metagenomic codes were designed to identify individuals based on specific microbial taxa or genes that are distinct and consistent across different body sites. Combining insights from microbial ecology and computer science, researchers discovered that it is possible to distinguish individuals from groups of hundreds based solely on their microbiomes, with over 80% accuracy even after a year, particularly notable in the case of the gut microbiome (Franzosa et al., 2015). While this underscores the fascinating individuality of human microbial signatures, it also raises significant privacy concerns for participants in microbiome research projects, highlighting the need for robust privacy safeguards in the handling of such health data (Chuong et al., 2017).

In Japan Tomofuji et al. (2023), uncovered a potential concern about metagenomic data obtained from human fecal samples. Specifically, they achieved a remarkable 93.7% accuracy in predicting biological gender by analyzing the read depth of non-pseudo-autosomal regions of sex chromosomes. This report has significant effects, especially in the context of human microbiome studies, where it can help rectify mislabelled samples and contribute to the field of human genetics. However, the accurate prediction of genetic sex bearing privacy concerns, particularly for individuals who may not wish to disclose this information. This concern is especially relevant to transgender individuals, who may face varying degrees of legal protection worldwide. To address these privacy issues, methods for removing human DNA reads from metagenomic data were developed during the National Institutes of Health's Human Microbiome Project (Wagner et al., 2016). It is worth noting that sex prediction based on DNA extracted from fecal samples had previously been predominantly conducted for wild animals using PCR amplification of marker genes (Guccione et al., 2023).

Furthermore, another study demonstrates sensitivity in identifying matched genotype data and accurately predicted ancestral backgrounds in samples. Ancestral backgrounds were defined as American, European, African, East Asian, and South Asian (Tomofuji et al., 2023). These findings highlight the importance of considering the ethical implications and privacy concerns when utilizing open-source microbiome data.

### 4.8 Improving metadata quality in microbiome research

Metadata is essential for the interpretation, reproducibility, and reuse of microbiome data. However, metadata quality is often variable, which can hinder research progress. To improve metadata quality, we can consider employing Manual and Automated curation. The first one is the most accurate approach, but it is also the most time-consuming and expensive. The latter employs ML approaches and other techniques to extract metadata from raw sample data. It is the most scalable approach, but it can be less accurate than the first one. One example of an automated curation approach is the ML framework developed by Nassar et al. (2022) that automatically extracts important metadata from a vast number of metagenomics studies found in the Europe PMC literature repository. This integration allows for the continual enhancement of current metadata in ENA and MGnify metagenomics studies by sourcing information from research articles. As a result, the MGnify database now displays these annotations, providing information on metadata like health status, disease conditions, geographic locations, and sequencing methods. Gonçalves and Musen (2019) study shed light on the varying quality of metadata available in prominent databases such as NCBI's BioSample and the European Bioinformatics Institute's BioSamples. One of the contributing factors to this variability is the infrequent use of controlled vocabularies during the metadata submission process. Additionally, the allowance for the creation of user-defined attributes has resulted in a proliferation of heterogeneity within the metadata landscape. This diversity often poses challenges for researchers, making it difficult to harness the full potential of information within a specific dataset or across multiple datasets (Gonçalves and Musen, 2019).

Klie et al. (2021) aimed to enhance the metadata coverage of SRA BioSample entries using deep learning-based named entity recognition (NER). The study achieved high prediction accuracies for certain metadata categories when extracting information from sample titles (TITLEs). It is worthy to note, they processed all the available BioSample up to May 2018, and Genus/Specie and strains generally refers to processed samples. However, lower accuracies and the absence of predictions for other metadata categories underscored existing issues with the current metadata annotations in BioSample. These findings demonstrate the effectiveness of recurrent neural networks for NER-based metadata prediction and suggest the potential of such models to expand metadata coverage in BioSample, reducing the reliance on manual curation (Klie et al., 2021). Below some additional thoughts on the future directions of machine learning for metadata retrieval in metagenomics. Firstly, ML algorithms (De et al., 2022; Nassar et al., 2022; Raghavendra Nayaka and Ranjan, 2023) could be developed to extract metadata from scientific literature, abstracts, and environmental monitoring data. This would allow researchers to extract more reliable metadata with less effort. Secondly, ML algorithms could be used to develop new metadata standards that are tailored to specific research questions. This would help to ensure that metadata is collected in a way that is most useful for the scientific community.

## 5 Metadata exploitation for robust ML models

During development of ML-based classifiers, the incorporation of metadata emerges as a crucial factor for accurate predictions and robust model development. A series of studies mark the significance of considering host associated metadata elements, ranging from geographical location to dietary habits and perinatal factors, host genetic factor (Lopera-Maya et al., 2022; New et al., 2022) shedding light on microbial compositions. Below we have highlighted examples of why researchers should consider host associated factors to train supervised predictive ML model for better generalization capability on the unseen dataset.

### 5.1 Changes in the gut microbiome: from infancy to adulthood and beyond

Studies have shown that the gut microbiome of infants undergoes significant changes during the first 3 years of life, with differences observed between populations and influenced by factors, such as delivery mode. Yatsunenko et al. (2012) compared fecal samples from Amerindians in Venezuela and residents of U.S. metropolitan areas, finding that the gut microbiome exhibited similar functional maturation patterns across the initial 3 years of life across populations. Palmer et al. (2007) also, observed substantial variation in the composition of gut bacteria in infants during the first year of life, with reduced variation within twin pairs and decreased variation with age. Orrhage and Nord (1999) emphasized the impact of delivery mode on the infant microbiome (Fanaro et al., 2003; Penders et al., 2006; Yatsunenko et al., 2012). Studies have shown that cesarean section (CS) results in a different microbiota compared to vaginal delivery (VD) (Bennet and Nord, 1987; Hällström et al., 2004; Elovitz et al., 2019). Cheng et al. (2022) emphasized the importance of further investigation to comprehensively delineate the multifaceted factors shaping microbiota dynamics during maternal-neonatal interactions, extending beyond traditional perinatal considerations.

Gudnadottir et al. (2022) employed the network-meta-analysis method and revealed that the microbiome demonstrates predictive potential for preterm birth and emphasizes the significance of specific microbial compositions in the vaginal microbiome as potential indicators for the likelihood of preterm birth.

Odamaki et al. (2016) and Meng et al. (2022) delved into the alterations in gut microbiota across different age groups and their associations with gut inflammation, particularly during the sexual maturity stage in healthy individuals. As individuals progress in age, there is a significant increase in the relative abundance of Firmicutes, accompanied by a concurrent decrease in the relative abundance of Bacteroides. The study further identified a positive correlation between body weight and the Firmicutes:Bacteroides ratio, shedding light on potential associations between microbiota composition and physiological parameters.

In addition to the age-related patterns identified in gut microbiota, the investigation also observed variations in microbial compositions across different body sites, including the vagina, skin, oral cavity, and respiratory tract. Detailed information on these variations is available at (Hou et al., 2022).

Kim et al. (2020) outlined that gender constitutes a significant variable shaping the composition of the gut microbiota. Furthermore, an investigation involving male and female germ-free C57BL/6J mice, Wang et al. (2016) and Zhao et al. (2019) revealed distinctive microbial preferences in the intestines of male and female mice. Despite these findings highlighting the relevance of gender in microbiota dynamics, a comprehensive understanding of this association remains elusive.

Cheng et al. (2022) emphasized geographical location as a paramount variable influencing the overall structure of maternal and neonatal microbiota, especially evident in two distinct populations from Asia and Europe. Elsherbiny et al. (2022) in Egypt elucidated the impact of geographical location on the gut microbiota in children with Type-1 Diabetes Mellitus, revealing differences in alpha diversity between controls and diabetic groups.

The Chinese healthy gut project (Ren et al., 2023), outlined on the correlation between gut microbiota and various dietary and lifestyle factors among healthy individuals in China. Notably, lifestyle phenotypes, including sleep procrastination, negative mood, and drinking habits, exhibited substantial influence on gut microbiota composition, with these factors showing the largest effect sizes.

#### 5.1.1 Role of diets

Noble et al. (2021) investigated the impact of sugar-sweetened beverage consumption during adolescence on the gut microbiome, which was linked to alterations in hippocampal function, as already demonstrated by David et al. (2014). Vujkovic-Cvijin et al. (2020) identified unexpected sources of gut microbiota variance, including alcohol consumption frequency and bowel movement quality. Singh and Mittal (2020) and Gacesa et al. (2022) comprehensively reviewed the profound impact of diet on the pathophysiology of mental disorders, highlighting its crucial role in shaping mental health outcomes. Ren et al. (2023) delved into the effects of dietary factors on the structure of the gut microbiota, while Manor et al. (2020) highlighted the composition-specific nature of host-microbe associations, providing insights into the intricate connections between microbiome composition, clinical markers, and lifestyle factors.

#### 5.1.2 Medication and antibiotic exposure

BMI and insulin level: Bäckhed et al. (2004) has illuminated a substantial connection between the gut microbiota and the regulation of body weight. Also, Ridaura et al. (2013) demonstrated weight gain in germ-free mice following gut microbiota transplants from individuals with obesity. These findings highlight the intricate relationship between gut microbiota composition and its role in regulating body weight. Gupta et al. (2020) emphasized the use of BMI scores to classify underweight, overweight, or obese individuals. Evans et al. (2014) shows that physical activity could shifts in the composition of the gut microbiome in animal models (Kang et al., 2014) but the robustness of this association at population-level remains uncertain. Concerning antibiotics, two cohort studies, utilizing a difference-in-differences approach, demonstrated that antibiotic exposure in infancy altered the relative abundance of off-target species and antibiotic resistance genes (Ramirez et al., 2020; Ribeiro et al., 2020; Lebeaux et al., 2022; Patangia et al., 2022).

In the realm of machine learning challenges, MetAML, an ML-based classifier, revealed variable results between prediction tasks, cautioning against potential overestimation of disease prediction due to confounding factors like active antibiotic treatment (Pasolli et al., 2016).

Abdul Rahman et al. (2023) developed supervised and unsupervised ML models to predict colorectal cancer using global dietary data, encompassing both younger and older adults from seven major countries (Canada, India, Italy, South Korea, Mexico, Sweden, and the United States) and diverse sociodemographic factors. Su et al. (2022) show that the limitation of using a combined public dataset did not specify the co-morbidities and antibiotics; thus, model performance depends on the exclusion of these metadata.

### 5.2 Future direction

Previous studies show that the composition of the human gut microbiome varies significantly among individuals. This variability suggests that incorporating metadata, including confounding factors and dietary information, into ML models is highly beneficial. Figure 1 illustrates a potential approach for integrating metadata information alongside microbiome features. This integrated analysis can lead to novel research questions, refine sample and feature selection, and improve the robustness of predictive statistical and ML models, e.g. develop ML model to predict the phenotype of a host organism. The interplay between ML and metadata is crucial for effective model implementation. Incorporating host metadata into microbiota studies can ensure that groups are well-matched, enhancing the reliability and reproducibility of studies investigating diseases or phenotypes associated with distinct pathological, physiological, lifestyle, or dietary traits.

## 6 Conclusion

Integrated metadata analysis is essential for maximizing the potential of ML and other advanced techniques in microbiome research. While recent advances in metagenomics, metabolomics, and metaproteomics have generated a wealth of publicly available data, its comprehensive utilization is hindered by several challenges, including the need for substantial time investments, accessibility issues with metadata, computational resource requirements, and the need for specialized bioinformatic expertise. As widely discussed in the previous sections, the inclusion of metadata information in ML models development is crucial to avoid erroneous outcomes. Metadata become essential to attenuate the negative impact of confounding factors, both technical and biological. Moreover, either when multi-omics data integration is considered, the inclusion of clinical metadata about enrolled subjects emerge as a source of knowledge leveraging the models accuracy, as demonstrated by Leung et al. (2022). Indeed, this review highlights the importance of integrated metadata analysis in microbiome research. By combining microbial data with sample-specific information, researchers can gain a deeper understanding of the microbial communities that inhabit the human body and their role in health and disease. This knowledge can be used to develop new diagnostic and therapeutic strategies. However, integrated metadata analysis is also challenging due to issues related to data management, computational demands, integration approaches, and the selection of appropriate analysis tools. To fully leverage the potential of integrated metadata analysis in microbiome research, it is essential to address these challenges through the development of new tools and resources, as well as the training of researchers in the necessary skills.

## Author contributions

BK: Conceptualization, Writing—original draft, Writing—review & editing. EL: Writing—review & editing. BF: Writing—review & editing, Conceptualization, Supervision, Writing—original draft. GP: Conceptualization, Funding acquisition, Supervision, Writing—review & editing.
